# Genetically determined Alzheimer's disease research advances: The Down Syndrome & Autosomal Dominant Alzheimer's Disease 2024 Conference

**DOI:** 10.1002/alz.70309

**Published:** 2025-07-02

**Authors:** Neus Falgàs, Lucia Maure‐Blesa, Beau Ances, Lisi Flores‐Aguilar, Sigan Hartley, Jason Hassenstab, M Florencia Iulita, Matthew Janicki, Katherine Koenig, Patrick Lao, Johannes Levin, Eric McDade, Laurent Meijer, Michael S. Rafii, Heather M. Snyder, Raquel Sánchez‐Valle, Juan Fortea, DSAD‐ADAD conference group, Jose Arriola Infante, Mirea Balasa, Isabel Barroeta, Nicolas Barthelemy R, Alexandre Bejanin, Bessy Benejam, Beatriz Bosch, Angela Bradshaw, Maria Carmona‐Iragui, Ann Cohen, Aina Comas Albertí, Lajos Csincsik, A. Claudio Cuello, Laura del Hoyo Soriano, Janna Dijkstra, Natalie Edwards, Sandra Giménez, Fernando Gonzalez‐Ortiz, Brian Gordon, Sara Gutiérrez Fernández, Benjamin Handen, Charlotte Jacob, Erik Johnson, Charlotte Johansson, Albert Lladó, Alberto Lleó, Samuel Morabito, Alejandra. O. Morcillo‐Nieto, Laia Montoliu‐Gaya, Michael Okafor, Agnes Pérez‐Millan, Marie Claude Potier, John Ringman, Íñigo Rodríguez‐Baz, Eric Rubenstein, Natalie S. Ryan, André Strydom, Lidia Vaqué‐Alcázar, Lisa Vermunt, Laura Videla Toro, Shahid Zaman

**Affiliations:** ^1^ Alzheimer's Disease and Other Cognitive Disorders Unit, Service of Neurology Hospital Clínic de Barcelona Fundació de Recerca Clínic Barcelona‐Institut d'Investigació Biomèdica August Pi i Sunyer University of Barcelona Barcelona Spain; ^2^ Memory Unit Department of Neurology Hospital de la Santa Creu i Sant Pau Biomedical Research Institute Sant Pau Sant Antoni Maria Claret Departamento de Medicina Universitat Autónoma de Barcelona Barcelona Spain; ^3^ Department of Neurology Washington University School of Medicine in St. Louis St. Louis Missouri USA; ^4^ Department of Pathology and Laboratory Medicine University of California Irvine California USA; ^5^ Waisman Center University of Wisconsin‐Madison Madison Wisconsin USA; ^6^ Altoida Inc. Washington District of Columbia USA; ^7^ Department of Disability and Human Development University of Illinois Chicago Chicago Illinois USA; ^8^ National Task Group on Intellectual Disabilities and Dementia Practices Rockport Maine USA; ^9^ Sorbonne Université Institut du Cerveau‐Paris Brain Institute ‐ ICM CNRS, APHP, Hôpital de La Pitié Salpêtrière, InsermParis, France Hôpital Pitié Paris France; ^10^ Imaging Sciences Imaging Institute Cleveland Clinic Cleveland Ohio USA; ^11^ Gertrude H. Sergievsky Center and Taub Institute for Research on Alzheimer's Disease and the Aging Brain College of Physicians and Surgeons Columbia University New York New York USA; ^12^ Department of Neurology Ludwig‐Maximilians‐University Munich Germany; ^13^ German Center for Neurodegenerative Diseases Munich Germany; ^14^ Munich Cluster of Systems Neurology (SyNergy) Munich Germany; ^15^ Perha Pharmaceuticals; Centre de Perharidy Hôtel de Recherche Roscoff France; ^16^ Alzheimer's Therapeutic Research Institute USC Keck School of Medicine San Diego California USA; ^17^ Division of Medical and Scientific Relations Alzheimer's Association Chicago Illinois USA; ^18^ Center of Biomedical Investigation Network for Neurodegenerative Diseases (CIBERNED) Madrid Spain; ^19^ Barcelona Down Medical Center Fundació Catalana de Síndrome de Down Entresòl Barcelona Spain; ^20^ Alzheimer Europe Bertrange Luxembourg; ^21^ Department of Psychiatry University of Pittsburgh Pittsburgh Pennsylvania USA; ^22^ School of Medicine, Dentistry and Biomedical Science Queen's University Belfast University Rd Belfast UK; ^23^ McGill University Department of Pharmacology and Therapeutics McGill University Montreal Quebec Canada; ^24^ Genomics of Neurodegenerative Diseases and Aging, Department of Human Genetics Amsterdam University Medical Center Amsterdam Netherlands; ^25^ Department of Neurology Vagelos College of Physicians and Surgeons Columbia University New York City New York USA; ^26^ Department of Neuroscience Columbia University New York City New York USA; ^27^ Multidisciplinary Sleep Unit. Respiratory department. Hospital de la Santa Creu i Sant Pau Biomedical Research Institute Sant Pau Barcelona Spain; ^28^ Department of Psychiatry and Neurochemistry Institute of Neuroscience and Physiology University of Gothenburg Gothenburg Sweden; ^29^ VIB‐KU Leuven Center for Brain & Disease Research VIB Center for Brain & Disease research Leuven Belgium; ^30^ Laboratory of Neurochemistry and Behaviour, Experimental Neurobiology Unit University of Antwerp Antwerpen Belgium; ^31^ Department of Neurology Emory University Atlanta Georgia USA; ^32^ Division of Neurogeriatrics Department of Neurobiology Care Sciences and Society Karolinska Institutet Solna Stockholm Sweden; ^33^ Department of Neurology University of Southern California Los Angeles California USA; ^34^ Department of Epidemiology Boston University Boston Massachusetts USA; ^35^ Dementia Research Centre Department of Neurodegenerative Diseases UCL Queen Square Institute of Neurology, Queen Square London UK; ^36^ Institute of Psychiatry, Psychology, and Neuroscience King's College London Strand London UK; ^37^ Neurochemistry Laboratory Department of Laboratory Medicine Amsterdam Neuroscience VU University Amsterdam UMC Amsterdam the Netherlands; ^38^ Department of Psychiatry University of Cambridge Cambridge UK

**Keywords:** Autosomal dominant Alzheimer's disease, Biomarkers, Clinical trials, Down syndrome, Neuroimaging, Pathophysiology

## Abstract

**INTRODUCTION:**

The Down syndrome‐associated Alzheimer's disease (DSAD) autosomal dominant Alzheimer's disease (ADAD) 2024 Conference in Barcelona, convened under an Alzheimer's Association International Society to Advance Alzheimer's Research and Treatment (ISTAART) grant through the Down syndrome and Alzheimer's disease (AD) Professional Interest Area (PIA), brought together global researchers to foster collaboration and knowledge exchange between the fields of DSAD and ADAD.

**METHODS:**

This article provides a synthesis review of the conference proceedings, summarizing key discussions on biomarkers, natural history models, clinical trials, and ethical considerations in anti‐amyloid therapies.

**RESULTS:**

A total of 211 attendees from 16 countries joined the meeting. Global researchers presented on disease mechanisms, therapeutic developments, and patient care strategies. Discussions focused on challenges and opportunities unique to DSAD and ADAD. Experts emphasized the urgent need for tailored clinical trials for ADAD and DSAD and debated the safety and efficacy of anti‐amyloid treatments. Ethical considerations highlighted equitable access to therapies and the crucial role of patient and caregiver involvement.

**DISCUSSION:**

The conference highlighted the importance of inclusive research and collaboration across the genetic forms of AD.

**Highlights:**

- Biomarker research and natural history models developed in Down syndrome‐associated Alzheimer's disease (DSAD) and autosomal dominant Alzheimer's disease (ADAD) enable the prediction of disease progression not only for DSAD and ADAD, but also for sporadic Alzheimer's disease (AD).
‐Collaboration and knowledge exchange among researchers across these genetic forms of AD will accelerate our understanding of the pathophysiology and advance preventive trials in DSAD and ADAD.
‐Tailored clinical trials for DSAD are urgently needed to address specific safety and efficacy concerns.
‐Inclusive research practices are crucial for advancing treatments and understanding of DSAD and ADAD.

## 1 INTRODUCTION

Autosomal dominant Alzheimer's disease (ADAD) and Down syndrome‐associated Alzheimer's disease (DSAD) are genetically determined forms of Alzheimer's disease (AD) characterized by unique clinical presentations and pathophysiological profiles that serve as crucial models for deepening our knowledge of AD. Studying these two conditions in tandem offers a unique opportunity to advance the field beyond what could be achieved by investigating them in isolation. While each disorder has distinct genetic origins, both ultimately converge on amyloid‐beta (Aβ) pathology, providing complementary insights into disease mechanisms, therapeutic targets, and potential intervention strategies.

ADAD accounts for less than 1% of all AD cases^1^ and is comprised of carriers of symptomatic mutations in genes such as amyloid precursor protein (*APP*), presenilin 1 (*PSEN1*), or presenilin 2 (*PSEN2*). The two latter code for the proteins PSEN1 and PSEN2, part of the γ‐secretase complex that is crucial for the cleavage of APP into beta‐amyloid Aβ. These mutations result in either an overproduction or an altered cleavage of amyloid‐beta (Aβ), ultimately driving the formation of AD neuropathological changes. Although ADAD mutation carriers typically exhibit early amnestic symptoms akin to sporadic cases, some may present additional behavioral and atypical manifestations with a very early onset. Despite its rarity, ADAD provides valuable insights into the underlying pathological processes of sporadic AD due to shared clinical manifestations and pathophysiology.^2^

DSAD, in turn, represents the most common genetic form of AD, with all individuals with complete trisomy of chromosome 21 developing AD neuropathological changes by the fourth decade and most developing symptomatic AD by the fifth to sixth decade.^3^, ^4^ However, diagnostic challenges arise in the earlier stages due to lifelong intellectual disability, which often overshadow AD‐related cognitive decline.^5^, ^6^ Indeed, the triplication of chromosome 21, including the *APP* gene, accounts for a ≈ 1.5‐fold increase in the abundance of APP mRNA levels in the brains of individuals with DS,^7^, ^8^ resulting in increased brain levels of Aβ and ultimately leading to the development of AD. Moreover, other genes of proteins involved in AD such as *ABCG1*, *SOD1*, *BACE2*, or *DYRK1A* among others are also found on chromosome 21, possibly impacting phenotypic variations.^9^

Overall, both ADAD and DSAD have served as cornerstones of the amyloid cascade hypothesis for AD (Hardy et al.^10^), strongly supporting the notion that the primary event in AD neuropathology is the formation of Aβ, whether through overproduction of APP or disruptions in its processing. Studying DSAD and ADAD together offers a powerful comparative framework to explore both overlapping and distinct pathological mechanisms, which may yield more robust biomarkers, refine therapeutic approaches, and enhance the generalizability of findings to sporadic AD.

Genetic forms of AD provide critical insights into its pathophysiology, revealing shared mechanisms and unique characteristics. Collaborative efforts are essential to foster cross‐disciplinary exchange, advance knowledge, and drive the design of preventive trials, with ADAD and DSAD representing ideal target populations^11^ (see Section 6.2). There is an urgent need to expand research efforts to better understand the disease processes underlying ADAD and DSAD.^12^ Such research will not only benefit these specific populations but also holds promise for broader therapeutic applications in sporadic AD. Establishing a dedicated scholarly platform for interdisciplinary collaboration is paramount to achieve these goals and accelerate progress in the field. By leveraging the complementary insights gained from studying both DSAD and ADAD, future research can drive scientific advancements that surpass what could be achieved by investigating these conditions independently. The DSAD‐ADAD conference aimed to bridge this gap by gathering international investigators to foster collaboration and knowledge exchange between the fields of DSAD and ADAD, with a focus on inclusivity and diversity. Its ambition is to become a notable milestone in the global discourse in genetically determined AD.

**RESEARCH IN CONTEXT**

1. **Systematic review**: We report the updates and advances in Down syndrome‐associated Alzheimer's disease (DSAD) and autosomal dominant Alzheimer's disease (ADAD) research presented at the DSAD‐ADAD Conference 2024 in Barcelona. This was the first dedicated conference to bring together researchers studying these two genetic forms of Alzheimer's disease (AD).
2. **Interpretation**: The conference highlighted significant similarities as well as differences between DSAD and ADAD. By exploring the central role of these genetic forms, along with their parallels and distinctions, this paper offers valuable insights to deepen understanding of AD.
3. **Future directions**: Further research is imperative to advance the understanding of ADAD and DSAD, with priorities focusing on disease pathophysiology, natural history, and the development of therapeutic strategies. Genetic forms of AD have been pivotal in shaping our knowledge and will continue to offer unique opportunities for deeper insight. A critical future direction, urged by both the scientific and patient communities, is the inclusion of these populations in therapeutic and preventive clinical trials. Initiatives like the DSAD‐ADAD Conference are key to fostering collaboration and research, generating novel hypotheses, sharing experiences, and advancing the field.

## 2 DSAD‐ADAD CONFERENCE STRUCTURE OVERVIEW

The convening of the DSAD‐ADAD Conference, held on April 11‐12, 2024 in Barcelona (Spain), addressed the unique challenges and opportunities posed by DSAD and ADAD.

The program was designed by a scientific committee composed of the Executive Committee of the Alzheimer's Association International Society to Advance Alzheimer's Research and Treatment (ISTAART) Down syndrome (DS) Professional Interest Area (PIA).^1^

The aim of the conference was to foster collaboration and disseminate knowledge among international experts, leveraging established networks such as the Dominantly Inherited AD Network (DIAN: https://dian.wustl.edu/), Alzheimer Biomarkers Consortium–Down Syndrome (ABC‐DS: https://abc‐ds.org/), Trial Ready Cohort for Down Syndrome (TRC‐DS: https://www.trcds.org/), the Horizon21 network (https://horizon‐21.org/) and the DSAD PIA within the ISTAART (https://istaart.alz.org/) network among other key stakeholders.

**FIGURE 1 alz70309-fig-0001:**
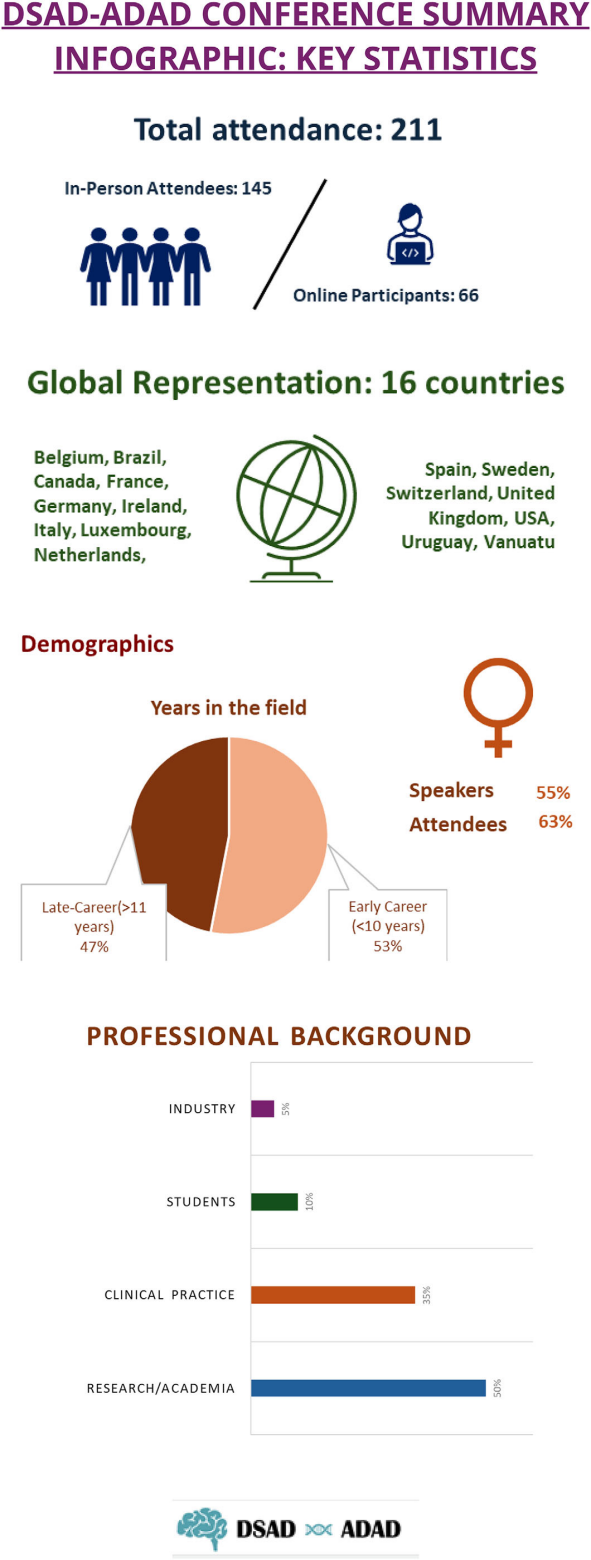
DSAD‐ADAD Conference summary infographic: Key statistics. ADAD, autosomal dominant Alzheimer's disease; DSAD, Down syndrome‐associated Alzheimer's disease.

The conference was structured in eight sessions as described in Table 1. The composition of each session was balanced to include established and emerging investigators with diverse expertise, who were asked to present five short talks (10–15 min) in each session, keeping almost 40% of the time for discussion. An additional session included “Flash‐talks”, where nine speakers presented a brief summary of their work. Further key statistics can be found in Figure 1.

**TABLE 1:** DSAD‐ADAD: Structure and aims of conference sessions

Session no.	Session title	Session aim/content	Session outcomes
1	Pathophysiology of DSAD and ADAD	This session focused on other AD biological and molecular pathways beyond the accumulation of amyloid and tau in these genetic forms.	Panelists noted several underscored specificities, including differences in cerebrovascular damage, inflammation, metabolic dysregulation, and genetic pathways.
2	Clinical picture	This session focused on advances regarding the clinical presentation of DSAD and ADAD.	Advances in the cognitive evaluation of individuals with DS, their temporal relationship with changes in biomarkers, and the clinical characterization of a new mutation in ADAD were highlighted.
3	Biomarkers	This session focused on advances in detecting AD and other co‐occurring conditions through different biomarkers in these genetic forms of the disease.	The panelists highlighted several advancements in AD biomarkers, including research on the latest CSF biomarkers, proteomics, and the detection of LBP, sleep disturbances, and epilepsy in these populations.
4	Neuroimaging	This session focused on the advances of neuroimaging as a biomarker to enable us to detect the disease.	Advances in molecular imaging techniques and structural and functional neuroimaging were underscored.
5	Natural history models	This session focused on natural history models of genetically determined forms of AD.	Panelists underscored recent large‐scale epidemiological data on DSAD and highlighted the possible inclusion of another form of genetically determined AD APOE4 homozygotes.
6	Unifying cohorts of genetically determined forms of AD and clinical trials	This session focused on the need to join forces to create collaborative research consortia and clinical trials specific to these populations.	Results were shown from previous single‐center and multicenter cohorts and previous and future clinical trials.
7	Collaboration and networking opportunities for AD research in individuals with Down syndrome and ADAD	This session focused on collaborative efforts and networking opportunities driving AD research in DSAD and ADAD.	Two main initiatives were discussed: the NIH INCLUDE project and Alzheimer Europe.
8	Ethical considerations and implications of AD research in individuals with DSAD and ADAD with the approval of new treatments	This session included a discussion with experts on the field and the audience regarding the ethical considerations of research and treatment among these populations, mainly DSAD.	The discussion focused on inclusion in specific DSAD trials and raised ethical and safety concerns shared among clinicians.

Abbreviations: AD, Alzheimer's disease; ADAD, autosomal dominant Alzheimer's disease; APOE4, apolipoprotein E4; CSF, cerebrospinal fluid; DSAD, Down syndrome‐associated Alzheimer's disease; LBP, Lewy body pathology.

The following sections synthesize key insights from both presentations and discussions from the conference, capturing the main scientific themes, debates, and emerging perspectives shared in the study of DSAD and ADAD.

## 3 PATHOPHYSIOLOGY OF DSAD AND ADAD

While DSAD and ADAD represent clear examples where amyloid accumulation is a known disease phenomenon, other mechanisms are increasingly gaining relevance in the pathophysiology of AD. Although the metabolism and accumulation of Aβ and phosphorylated tau protein remain essential aspects, additional factors such as cerebrovascular pathology, inflammation, metabolic dysregulation, and genetic pathways are playing a growing role in understanding the disease.^13^ The first session underscored several of these specificities, highlighting their potential to lead to meaningful discoveries about the natural history of AD.

### 3.1 Vascular changes in DSAD and ADAD

The frequent presence of Aβ deposition in the cerebral vessel walls, known as cerebral amyloid angiopathy (CAA), in the brains of individuals with AD is increasingly recognized as part of the disease continuum.^14^ Indeed, upon cleavage of APP by *γ*‐secretase, two major Aβ species are produced: the less soluble Aβ1‐42, which forms extracellular fibrils and aggregates in the parenchyma, later forming the major part of the amyloid plaque; and the more soluble Aβ1‐40, which preferentially deposits in the walls of brain vessels and leptomeninges, leading to CAA. Its accumulation may indicate an imbalance in Aβ production and clearance, and it may remain undetected or appear as hemorrhagic (e.g., lobar hemorrhages, microbleeds, cortical siderosis) or non‐hemorrhagic (e.g., white matter hyperintensities [WMH], microinfarcts, inflammation) findings in neuroimaging,^15^, ^16^, ^17^, ^64^ sometimes accompanied by clinical symptoms. This vascular pathology occurs in all forms of AD but has been previously shown to be more prominent in genetic cases.^18^ As individuals with DS have low rates of other systemic vascular risk factors, the study of vascular changes among this group represents a unique opportunity to enlarge our understanding of the natural history of these disorders uniquely related to the AD pathophysiology.^19^, [Fig alz70309-fig-0001]

Research on *post mortem* analyses of advanced DSAD brain tissues has revealed significant vascular remodeling, including increased vessel length, higher pericyte density, collapsed capillaries, and fibrin deposition, suggesting compromised blood–brain barrier (BBB) integrity and vascular morphological changes. These findings could potentially mark a sequence of events from parenchymal Aβ accumulation to vascular Aβ deposition and BBB damage.

Parallel investigations^20^ have explored Aβ and tau pathology across different genetic and sporadic forms of AD. Individuals with APP duplications (APPdup), APPV717 mutations, DSAD, sporadic AD, and young individuals with DS were analyzed, revealing distinct patterns of parenchymal and vascular Aβ deposition. APPdup and DSAD cases showed higher vascular Aβ deposits than parenchymal deposits, contrasting with the predominant parenchymal Aβ burden in sporadic AD and APPV717 mutation carriers. Interestingly, the amount and localization of Aβ deposits among carriers of three copies of the *APP* gene (including individuals with DSAD and *APPdup*) varied, suggesting other factors apart from the *APP* gene dose may play a role and potentially pointing to protective mechanisms in individuals with DSAD.

The temporal relationship between parenchymal and vascular Aβ deposition remains a critical question. In DSAD and APPdup, vascular deposits were consistently associated with some degree of parenchymal involvement, suggesting a progression from parenchymal accumulation to vascular deposition.

Understanding these mechanisms could refine therapeutic approaches, particularly regarding the cerebrovascular implications of anti‐amyloid immunotherapies. Indeed, higher CAA burden in APPdup and DSAD individuals raises concerns about potential amyloid‐related imaging abnormalities (ARIA) in populations undergoing treatment. However, it was noted that tissue samples from pre‐dementia DS individuals show no vascular Aβ deposits,^20^ supporting early intervention to mitigate risks. Moreover, protective factors encoded on chromosome 21 may further differentiate DSAD from APPdup in terms of vascular pathology, offering a promising avenue for biomarker discovery and personalized therapy.

In summary, these investigations underscore the complexity and represent significant advancements in understanding the role of CAA in AD, particularly in genetic AD. Given the implications for anti‐amyloid therapies, continued collaborative research in this area is essential to developing effective treatments and improving patient care.

### 3.2 Inflammation and metabolic dysregulation

While the amyloid cascade and tau pathology have long dominated the understanding of AD, recent research highlights the significant role of neuroinflammation and its interaction with these hallmark features as a key driver in the progression of AD.^21^

Microglial activation, a hallmark of neuroinflammatory responses in AD, has been closely linked to Galectin‐3 (Gal‐3), a lectin‐family protein involved in inflammation and immune regulation.^22^ Elevated levels of Gal‐3 in cerebrospinal fluid (CSF) and brain tissue, particularly in neurodegenerative disorders such as frontotemporal dementia (FTD), have drawn attention to its role as a potential mediator of neurodegeneration.^23^ Extending this research to ADAD, increased Gal‐3 levels in neocortical tissue have been associated with cortical thickness changes, suggesting that Gal‐3‐mediated microglial activation could contribute to the inflammatory cascade driving disease progression. These findings position Gal‐3 not only as a potential biomarker but also as a therapeutic target in both genetic and sporadic AD, warranting further exploration of its mechanistic role in the disease.

In parallel, studies on nerve growth factor (NGF) dysregulation have provided a deeper understanding of how inflammation interacts with neurotrophic pathways in AD. Recent research has challenged the assumption that mature NGF (mNGF) is directly released in the brain, revealing instead that proNGF undergoes extracellular conversion via a cascade of zymogens, convertases, and inhibitors.^24^ This process is disrupted in both sporadic AD and DSAD, leading to proNGF accumulation and mNGF depletion. The resulting loss of trophic support to basal forebrain cholinergic neurons accelerates their atrophy and contributes to early cognitive decline.^25^ NGF dysregulation is further linked to amyloid burden, and its detection in tissue and fluid biomarkers offers promising diagnostic and therapeutic avenues for addressing this critical aspect of AD pathology.^26^

Moreover, and related to the previous section, the interaction between neuroinflammation and cerebrovascular pathology has emerged as a pivotal area of investigation. In DSAD, previous studies have shown a unique neuroinflammatory phenotype consistent with immune activation due to invasion of serum proteins in the brain,^27^ thus possibly relating to CAA and BBB damage. More recent studies align with these findings, showing that WMH, a marker of CAA, and glial fibrillary acidic protein (GFAP), a marker of astrocytosis and neuroinflammation, are independently associated with progression from mild cognitive impairment (MCI) to dementia.^28^ This reinforces the interplay between vascular pathology and neuroinflammation in driving AD progression and highlights the need to explore these processes as interconnected contributors to the disease's natural history.

### 3.3 Gene expression

Advancing our understanding of gene expression in genetically determined AD provides valuable insights into the molecular pathways that drive disease progression and enables the identification of potential biomarkers and therapeutic targets. Recent research has identified key genes with differential expression patterns that may serve as early indicators of disease and reveal novel aspects of AD pathogenesis.

One study focused on whole‐blood gene expression in ADAD and highlighted consistent upregulation of FXYD5 and MAPT in both symptomatic and asymptomatic mutation carriers. The differential expression of these genes, detectable even in the preclinical stages, positions them as promising biomarkers for ADAD. Other genes, including APP, PSEN1, SCARNA2, and TMEM184B, also exhibited significant expression changes, further delineating the transcriptomic landscape of ADAD and its potential molecular drivers.

Expanding on these findings, transcriptome studies comparing genetic and sporadic forms of AD have shed light on both heritable and environmental factors influencing disease progression.^29^

Additionally, emerging research has begun exploring the relationship between molecular variations in Aβ profiles linked to specific ADAD mutations and the age of disease onset.

Together, these findings underscore the value of gene expression and transcriptomic studies in unraveling the complexities of AD. They open new avenues for the development of biomarkers that can facilitate early detection and personalized interventions, as well as provide a foundation for investigating the interplay of molecular, genetic, and environmental factors in AD pathophysiology.

## 4 CLINICAL PICTURE

The second session of the conference highlighted advancements in understanding the clinical picture of AD in these genetic forms.

In DSAD, these studies explored the challenges of early diagnosis, the timing of cognitive decline, the heterogeneity of clinical presentations, and the influence of intellectual disability and cognitive reserve in this high‐risk population.

Biomarker‐based approaches have proven instrumental in predicting cognitive outcomes. Using the AT(N) framework, research demonstrated that biomarker status significantly predicts cognitive decline and the likelihood of transitioning to MCI or dementia in adults with DS. Moreover, research has shown that cognitive decline typically begins approximately 2.7 years after individuals become amyloid‐positive on positron emission tomography (PET) imaging, this insight being crucial for the design of interventions and clinical trials targeting DSAD and offering a clearer window for therapeutic action (see Section 6.2 on clinical trials).

Regarding the clinical presentation, large‐scale studies have underscored the nearly full penetrance of AD dementia in individuals with DS and the feasibility of longitudinal cognitive assessments to detect cognitive decline among this population.

Furthermore, case studies have demonstrated clinical heterogeneity in these populations, with atypical AD presentations, such as posterior cortical atrophy.^30^ While these cases emphasize a broadening spectrum of clinical features seen in DSAD (and ADAD), their relative rarity (compared to sporadic AD), might provide hints to the pathophysiological mechanisms underlying atypical AD presentations.

Finally, generational changes in the intellectual abilities of individuals with DS have also been a subject of exploration. Enhanced community support and enriched environments over recent decades have led to improved intellectual profiles and functionality in adults with DS. However, despite these generational shifts, evidence for a cognitive reserve effect influencing AD presentation remains limited. Collaborative initiatives, such as the CROSS‐PIA program within ISTAART, aim to bridge these gaps by integrating research on DS and resilience factors.

Moreover, in ADAD, recent findings have shed light on the A431E substitution in PSEN1, a mutation identified in the Los Altos region of Jalisco State. This mutation is associated with ADAD, characterized by significant spasticity early in the disease course, severe CAA pathology, and distinct tau deposition patterns. Studies have revealed that this mutation is present in at least 100 families, with an estimated 1260 individuals at risk. These insights highlight the unique clinical and pathological features linked to this specific genetic variant.

## 5 BIOMARKERS

Biomarker research has assumed paramount importance among ADAD and DSAD due to these conditions' unique genetic underpinnings and early onset characteristics. While traditionally focused on key pathophysiological markers such as Aβ and tau proteins, which remain a pivotal area of interest, recent advancements have also integrated other fluid and neuroimaging candidates as well as findings from proteomics and other innovative technologies that aid in early diagnosis and tracking of disease progression.

### 5.1 Fluid AD biomarkers (CSF and plasma)

Tau pathology has been particularly well‐characterized through studies of CSF biomarkers in ADAD. Changes in phosphorylated tau (p‐tau) residues such as 217, 181, 205, and 202, as well as MTBR‐tau243, have emerged as critical indicators of disease progression.^31^, ^32^ Notably, MTBR‐tau243 demonstrates significant changes closer to symptom onset, making it a particularly precise marker for tracking late preclinical and early clinical phases. Plasma p‐tau217, while applicable beyond ADAD, has shown utility in mirroring amyloid PET in preclinical stages and tau PET in symptomatic stages, offering further insights into its role as a biomarker.

Importantly, the study of fluid biomarkers in ADAD mutation carriers has highlighted the heterogeneity of mutations and their variable impacts on biomarker expression and disease progression.^33^

In addition to tau markers, GFAP has emerged as a promising indicator of preclinical disease activity in ADAD. Rising approximately a decade before symptom onset, GFAP levels are thought to reflect amyloid toxicity and neuroinflammatory processes, positioning it as a valuable tool for tracking early disease dynamics.^34^, ^35^

Moreover, research into biomarker correlations with neuropsychiatric features of DS has expanded the scope of biomarker applications. For instance, the Behavioral and Psychological Symptoms of Dementia in DS (BPSD‐DS II) scale has linked these symptoms to distinct biomarker patterns, improving understanding of the neuropsychiatric manifestations associated with dementia in this population.

### 5.2 Proteomic and molecular studies

Recent research has leveraged cutting‐edge proteomic and molecular approaches to uncover the mechanisms underlying AD pathogenesis. By studying proteomic changes occurring in individuals with DS that lead to DSAD and comparing them with those in other forms of the disease‐including ADAD and late‐onset AD (LOAD), studies find common alterations but unique temporal patterns between the groups. Characteristically, in DS, most proteomic changes occurring prior to the onset of AD symptoms involve early elevations in the levels of immunoglobulins, complement, collagen, and extracellular matrix proteins, as well as early decreases in synaptic proteins, neurofilament light, and lactadherin – a proposed protein marker for CAA.

Interestingly, and discussed throughout the conference, these changes suggest the loss of BBB integrity and cerebrovascular dysfunction as important early pathological changes in DSAD.

Expanding the scope of AD research, molecular investigations have also examined the influence of cholesterol on Aβ production via APP endocytosis. Building on prior evidence linking cholesterol to AD pathology,^36^ a collaborative study utilizing CRISPR/Cas9 technology explored the K28A mutation in the cholesterol‐binding domain of APP. Results showed that the K28A mutation produces shorter, non‐toxic Aβ peptides, even when combined with the familial London mutation. These findings suggest that targeting APP‐cholesterol interactions could serve as a therapeutic strategy to mitigate Aβ toxicity in AD.

### 5.3 Detection of co‐pathologies and co‐occurring medical conditions

Recent research has focused on the presence of co‐existing pathologies and comorbidities in AD, particularly in genetically determined forms, highlighting the intricate interplay of various pathophysiological factors, including Lewy body pathology (LBP), sleep disturbances, and epilepsy, as contributors to disease progression and clinical manifestations.

LBP is a common co‐pathology in AD,^37^ and recent work has sought to extend this understanding to genetically determined AD. Using an α‐synuclein seed amplification assay (SAA) in CSF, researchers are examining the timing, prevalence, and clinical implications of LBP across genetic and sporadic AD cohorts.

Sleep disturbances, increasingly recognized as both symptoms and potential risk factors for AD, are particularly prevalent in individuals with DS. Over 75% of cognitively unimpaired adults with DS have obstructive sleep apnea (OSA), a condition known to accelerate cognitive decline and worsen as AD progresses.^38^ Studies in DSAD have revealed impaired sleep efficiency, reduced rapid eye movement (REM) sleep, and heightened OSA severity compared to non‐demented individuals with DS, highlighting the urgent need for routine sleep screenings and interventions to mitigate cognitive decline.

Epilepsy also emerges as an important comorbidity, particularly in genetic forms of AD. Data show an earlier and higher prevalence of epilepsy in ADAD mutation carriers^39^ and DSAD, where a specific term has been coined: Late‐Onset Myoclonic Epilepsy in Down Syndrome (LOMEDS).^40^ This highlights the importance of early detection and targeted treatment for epilepsy as part of comprehensive care for AD patients. Additionally, electroencephalogram (EEG) microstates have been identified as potential markers for disturbed temporal dynamics in AD, offering a promising tool for monitoring disease progression.

## 6 NEUROIMAGING

Neuroimaging has become an essential tool in diagnosing and monitoring AD, particularly valuable as a biomarker. These studies enable a closer understanding of the natural history of the disease, including the timing, magnitude, and spatial distribution of cerebrovascular disease, amyloid accumulation, tau spread, hypometabolism, cortical thinning, and atrophy. In the following sections, we will cover the neuroimaging topics covered during the conference.

### 6.1 Amyloid and tau PET

Recent advances in tau and amyloid PET imaging have deepened our understanding of AD pathology in genetically determined populations. While amyloid PET imaging reveals overall similar patterns of amyloid accumulation in these groups,^41^ tau PET imaging highlights some differences in the spatial distribution, magnitude, and temporal progression of tau pathology.^42^

Comparative studies show that the general progression of amyloid accumulation, followed by tau deposition, is consistent across DSAD and ADAD. However, tau PET imaging reveals that individuals with DSAD exhibit greater tau levels for a given level of amyloid compared to ADAD. Additionally, the timing of tau accumulation appears to be more closely aligned with amyloid elevations in DSAD, suggesting a faster coupling of these pathologies relative to ADAD.^43^

Spatially, tau burden differs between the two groups. In DSAD, tau deposition is most prominent in subcortical and medial temporal regions, whereas ADAD shows earlier and more widespread tau accumulation in the parietal cortex and temporal lobe. These regional differences in tau distribution may reflect unique underlying biological drivers of pathology, shaped by the genetic and developmental context of each population.

The differences in the timing and spatial pattern of tau accumulation relative to amyloid provide critical insights for tailoring clinical trials and treatment strategies. For instance, the accelerated temporal coupling of amyloid and tau in DSAD aligns with previous commented results (Section 2) showing that in DSAD, cognitive decline starts 2.7 years after amyloid PET positivity, suggesting that therapeutic interventions targeting tau pathology may need to be initiated earlier in this group. Additionally, understanding the distinct regional patterns of tau deposition could inform the development of population‐specific diagnostic criteria and therapeutic targets.

### 6.2 Hypometabolism: Fluorodeoxyglucose‐PET (FDG‐PET)

In the natural progression of AD, hypometabolism in specific brain regions follows amyloid and tau pathologies and occurs before the onset of cognitive decline.^44^ In sporadic AD, this characteristic hypometabolic pattern, primarily involving lateral and parietal regions, is a well‐established marker for distinguishing MCI due to AD from other causes. Similar patterns are emerging in individuals with DS^5^, ^45^, ^65^ where hypometabolism is negatively associated with age and more pronounced in individuals with DSAD, particularly in the temporal and parietal regions, closely mirroring the patterns observed in sporadic AD. This reinforces the similarities between genetic and sporadic forms of AD.

### 6.3 Structural MRI and cerebrovascular disease markers

Recent advancements in MRI techniques have provided critical insights into cortical thickness, atrophy patterns, and cortical asymmetry, offering a detailed view of the structural changes associated with AD. Moreover, neuroimaging is also appearing as a powerful tool in the early detection of cerebrovascular disease, as well as novel techniques that were presented during the conference.

#### 6.3.1 Medial temporal lobe atrophy in DSAD

Medial temporal lobe (MTL) volumes are well‐established biomarkers for detecting early AD‐related changes, especially in DSAD. Studies consistently highlight progressive atrophy in MTL subregions, including the hippocampus, posterior parahippocampal gyrus, and entorhinal cortex, as individuals with DS age.^46^, ^47^ Notably, the entorhinal cortex and posterior hippocampus exhibit the earliest decline, occurring approximately 13–15 years before the estimated years to symptom onset (EYO),^4^ underscoring the critical role of MTL subregions as early indicators of neurodegeneration in DSAD.^66^

#### 6.3.2 Cortical thickness patterns in ADAD and DSAD

Cortical thickness studies have revealed distinct structural changes in DSAD and ADAD, moving beyond traditional hippocampal measures to enhance early detection of AD‐related changes. In DSAD, widespread cortical thinning in parietal, temporal, and occipital regions correlates with amyloid accumulation and cognitive impairment.

In ADAD, cortical asymmetry has emerged as a promising biomarker for disease progression. The novel Cortical Asymmetry Index (CAI) has shown heightened asymmetry in asymptomatic and symptomatic mutation carriers compared to healthy controls. These findings correlate with disease severity metrics such as EYO, Mini‐Mental State Examination (MMSE) scores, and serum neurofilament light chain (sNfL) levels, indicating the potential utility of CAI for tracking disease progression.^48^

#### 6.3.3 Cerebrovascular alterations in DSAD

MRI studies have also highlighted cerebrovascular disease as a critical component of AD pathology in DS populations. Key markers include WMH, enlarged perivascular spaces (PVS), cerebral microbleeds, and cortical infarcts, which escalate with AD progression.^49^ Temporal analyses reveal that cerebrovascular changes begin in the early 30s,^19^ with infarcts and PVS followed by microbleeds, WMH, and amyloid deposition in both vasculature and parenchyma by the mid‐30s. Neurofibrillary tangle pathology (Braak stages I‐II) appears concurrently, progressing to later stages by the late 30s. Notably, WMH accumulation occurs more rapidly in women during later stages and in apolipoprotein E4 (APOE4) carriers at earlier stages, emphasizing the interplay of genetic and demographic factors in cerebrovascular pathology.

Moreover, other tools for the detection of vascular pathology among DSAD are emerging. Preliminary findings indicate that retinal and choroidal assessments could detect subtle vascular changes linked to CAA and AD‐related pathology.^50^

## 7 NATURAL HISTORY MODELS

This section delved into the latest findings from studies exploring natural history models in genetic forms of AD, highlighting epidemiological trends and genetic influences shaping AD in these vulnerable groups.

### 7.1 DSAD

Recent large population‐level studies^51^ have reinforced the significant prevalence of DSAD, with incidence rates estimated at 22.4 cases per 1000 person‐years. Epidemiological findings reveal earlier onset of DSAD among Hispanic, Native, and mixed‐race populations compared to white non‐Hispanic individuals. These findings align with data from smaller cohort studies, such as those by McCarron et al., Fortea et al., and Iulita et al.,^4^, ^5^, ^52^ and emphasize the influence of genetic, environmental, and socioeconomic factors in disease progression. It further underscores the critical need for targeted healthcare interventions and prioritized care, as DSAD has emerged as a leading cause of mortality among individuals with DS. These findings call for expanded efforts in epidemiological research and clinical care to address the high prevalence and demographic disparities in DSAD, advancing equitable access to diagnosis and treatment for this vulnerable population.

### 7.2 APOE4 homozygotes: A new type of genetic AD?

Recent research has challenged the traditional view of APOE4 homozygotes as merely high‐risk individuals for AD, instead proposing their classification as a distinct genetic form of the disease. A large‐scale study involving 3297 individuals for pathological analysis and 10,039 for clinical evaluation revealed that nearly all APOE4 homozygotes exhibited AD pathology, with elevated biomarkers starting as early as age 55. By age 65, almost all showed abnormal amyloid levels in CSF, and 75% had positive amyloid PET scans, demonstrating near‐complete penetrance of AD pathology in this group.^53^

These findings have significant implications for AD research and clinical practice. The distinct biomarker trajectories and pathological features of APOE4 homozygotes highlight the need for tailored prevention strategies, innovative clinical trials, and personalized therapeutic approaches. Recognizing APOE4 homozygosity as a genetic subtype of AD could reshape the understanding of genetic risk, enabling earlier detection and more targeted interventions for this population.

The conference discussions supported this reconceptualization, emphasizing its potential to advance the study of genetic risk factors in AD. By framing APOE4 homozygotes as a unique genetic category, the field is poised to address the complexities of APOE4‐driven pathology more effectively and to leverage this understanding for improved patient outcomes.

## 8 STANDARDIZING COHORTS OF GENETICALLY DETERMINED FORMS OF AD AND CLINICAL TRIALS

Disease progression models in genetic forms of AD provide an essential roadmap for prevention and treatment trials. The past decade has seen unprecedented successes in creating large observational cohorts and international consortia to understand the disease in these populations and, ultimately, offer them preventive treatments.

### 8.1 Single‐center cohorts

The Down Alzheimer Barcelona Neuroimaging Initiative (DABNI) cohort was started in Barcelona in 2014 from a collaboration agreement between Hospital Sant Pau and the Fundación Catalana de Síndrome de Down. It started with the creation of a highly specialized Down Syndrome Alzheimer Unit where all adults with DS were offered a free health plan, including complete neurological and neuropsychological assessments as well as blood tests when needed, personalized follow‐up, telematic support with a general practitioner, cognitive stimulation workshops, and support, training, counseling, and orientation for the caregivers. In parallel, every participant was offered the possibility to participate in the DABNI cohort and in research projects. From 2014 until March 2024, they have recruited 1,136 patients, from whom more than 1715 blood samples and 360 CSF samples have been collected and 467 MRIs, 154 amyloid PETs, 187 FDG PETs, and 60 Tau PETs have been performed. Moreover, 459 sleep studies have been conducted, and eight brains have been donated and analyzed.

The Barcelona‐ADAD cohort, a subset of the Genetic Counseling and Research Program for Familial Dementias (PICOGEN) which started in 2001 and is still ongoing^54^, ^55^ at the Hospital Clínic de Barcelona. The PICOGEN program includes not only ADAD families, but also genetic FTD and genetic prion diseases. Its primary objectives are the clinical and biological characterization of both symptomatic and asymptomatic ADAD mutation carriers.^55^, ^56^, ^57^ This observational study includes 61 participants, including presymptomatic mutation carriers, symptomatic mutation carriers, and non‐carrier familial controls. Participants undergo baseline assessments with optional follow‐up visits every 2 years, encompassing detailed cognitive evaluations and biomarker analyses. The study aims to differentiate ADAD from age‐related dementias, identify prognostic markers influencing disease severity, and explore potential therapeutic targets through collaborations with the DIAN Trials Unit (DIAN‐TU) and other international initiatives.

### 8.2 Multicenter collaboration and clinical trials

The conference emphasized that cross‐country collaboration is needed for harmonizing research protocols and ultimately for creating large‐scale clinical trials tailored to these specific populations.

#### 8.2.1 DSAD

Advances in DSAD research and clinical trials have been driven by several international consortia. The Horizon21 consortium has unified 13 sites across nine European countries to standardize procedures, enhance data sharing, and improve understanding of the mean age of AD diagnosis in DS.^58^ Efforts include refining cognitive outcome measures, such as CAMCOG‐DS‐II, currently validated in a multi‐country study involving over 220 participants.^59^

The Down Syndrome Biobank Consortium has created a harmonized international repository for tissues and biofluids from DSAD patients and controls, supporting more consistent research. Meanwhile, the Alzheimer's Clinical Trial Consortium in Down Syndrome (ACTC‐DS), funded by the National Institutes of Health (NIH) INCLUDE Initiative, has expanded to 20 sites across the United States and Europe. Its TRC‐DS includes approximately 230 participants undergoing neuroimaging, biomarker testing, and cognitive assessments.

Clinical trials targeting DSAD are progressing, with therapeutic approaches including immunotherapy, monoclonal antibodies, and antisense oligonucleotides against APP. ACI‐24.060, an enhanced vaccine, is in a phase 1b/2 trial across 14 sites. Other trials on monoclonal antibodies and ASO therapies are forthcoming.

Next year, a preventive trial on levetiracetam (LEV) will assess its safety and efficacy in preventing tonic‐clonic seizures in DSAD patients without epilepsy. This 2‐year study will monitor biomarkers and seizure incidence. Additionally, leucettinib‐21, a DYRK1A inhibitor.,^60^, ^61^ is in phase 1 trials to address cognitive deficits associated with DSAD by targeting overactive DYRK1A kinase, with plans to progress to phase 2A.^59^, ^62^

While there is great optimism for the future, particularly with the introduction of several new clinical trials for DSAD, significant barriers remain in improving recruitment and retention in these studies. During the conference, the importance of addressing these challenges was highlighted, emphasizing the need for greater awareness and engagement.

In DSAD, fostering education and dissemination of information is essential—not only among families and caregivers but also within the global medical and scientific community. A deeper understanding of this condition and its prevalence is key to driving both the development of clinical trials and participation in them. Knowledge empowers families, enabling informed decision‐making and advocacy for research that addresses their specific needs. At the same time, increasing awareness within the broader medical community enhances recognition of DSAD, encourages scientific engagement, and promotes the creation of tailored therapeutic and preventive trials. Despite recent progress, further efforts are needed, as expanding knowledge remains a crucial step toward advancing research and improving outcomes for individuals with DSAD.

#### 8.2.2 ADAD

In the ADAD field, The DIAN consortium leads global efforts in research, advancing understanding and treatment through three key initiatives. The DIAN Expanded Registry (DIAN EXR) connects affected families and researchers, facilitating collaboration and access to observational studies, clinical trials, and educational resources.

The DIAN‐TU conducts innovative clinical trials, testing multiple therapies simultaneously to enhance efficiency. Since 2012, trials have included drugs like gantenerumab, solanezumab, and tau‐targeting therapies such as E2814, often combined with open‐label lecanemab treatment.

The DIAN Observational Study, tracks disease progression in families with ADAD mutations to identify early biomarkers and inform prevention strategies. This work aligns with other groundbreaking research efforts, such as studies on the Colombian kindred, a large family in Antioquia, Colombia, with the PSEN1 E280A mutation that provided critical insights into disease progression and prevention, including identifying genetic factors like the APOE3 Christchurch variant that may delay symptom onset.^63^

DIAN's ongoing trials, including ART, Tau NexGen, and a Primary Prevention Trial, reflect its commitment to precision medicine and transformative treatments for ADAD.

## 9 COLLABORATION AND NETWORKING OPPORTUNITIES FOR AD RESEARCH IN INDIVIDUALS WITH DS AND ADAD

During the conference, there was a significant focus on collaborative efforts and networking opportunities driving AD research in individuals with DSAD and ADAD. Standout initiatives discussed included the NIH INCLUDE Project and Alzheimer Europe.

The NIH INCLUDE Project, launched in 2018 with $348 million in funding, addresses the health and quality‐of‐life needs of individuals with Down syndrome. It focuses on targeted basic science, biomarker evaluation, and clinical trials for DS, fostering collaboration through a data‐coordinating platform and funding high‐risk, high‐reward studies.

In parallel, Alzheimer Europe unites 41 national associations across 37 countries, advocating for dementia research and care. It emphasizes public involvement, ethical considerations, and support for caregivers, hosting its first collaborative brain health event in 2023 to integrate patient and caregiver perspectives into research.

Together, initiatives like INCLUDE and Alzheimer Europe showcase the power of collaborative, patient‐centered approaches to advance understanding, care, and prevention of AD. These initiatives highlighted the transformative impact of collective action in advancing research and care for these genetic forms of AD and underscored the necessity of engaging patients and their families throughout the research process, fostering inclusive approaches that prioritize their perspectives and needs.

## 10 ETHICAL CONSIDERATIONS AND IMPLICATIONS OF AD RESEARCH IN INDIVIDUALS WITH DSAD AND ADAD WITH THE APPROVAL OF NEW TREATMENTS

The exclusion of DSAD patients from trials of anti‐amyloid therapies, which have primarily focused on typical AD populations, raises ethical concerns about equitable access to these treatments. Questions persist about the safety and efficacy of these therapies for DSAD, a population often characterized by higher rates of CAA and variability in disease severity. This lack of representation complicates informed decision‐making and highlights the need to ensure potential benefits outweigh risks for DSAD patients. Insights from other populations, such as ADAD, can provide valuable guidance in tailoring safe and effective strategies for DSAD.

The limited number of dedicated clinical trials for DSAD underscores the importance of conducting targeted research to generate necessary data on treatment safety and effectiveness. Perspectives from patients and families reveal a willingness to participate in trials, emphasizing the urgency of addressing these gaps. Additionally, education and clear communication are essential for empowering patients and caregivers to make informed decisions about therapies and participation in research.

While challenges remain, experts agree that disease‐modifying therapies could significantly benefit DSAD populations. However, clinical trials specific to DSAD are vital before these treatments can be widely implemented. Inclusive engagement with patients, families, and caregivers is crucial to ensuring their voices shape the research and treatment landscape. Inspired by progress in ADAD through collaborative efforts, the scientific community must now take swift action to advance equitable access to transformative treatments for DSAD, avoiding unnecessary delays in providing these potentially life‐changing therapies.

## 11 CONCLUSIONS

**FIGURE 2:**
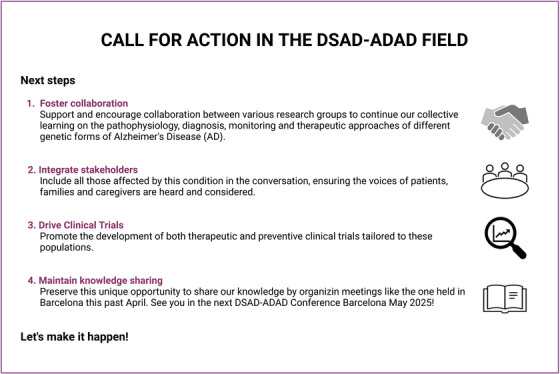
Call for action in the DSAD‐ADAD field. Created in https://BioRender.com. ADAD, autosomal dominant Alzheimer's disease; DSAD, Down syndrome‐associated Alzheimer's disease

The DSAD ADAD 2024 conference in Barcelona highlighted significant strides in biomarker research and biological models to better understand DSAD and ADAD‐determined AD forms. Critical discussions focused on key areas of overlap and divergence in DSAD and ADAD, while advancing early detection methods and monitoring disease progression using biomarkers. Ethical debates centered on equitable access to emerging therapies, particularly addressing safety concerns in DSAD due to higher amyloid angiopathy rates. Collaboration across disciplines and international borders emerged as essential for tackling the complexities of genetic AD. The conference underscored the need for tailored clinical trials, integrating patient perspectives to optimize treatment strategies and ensure ethical rigor in research (Figure 2).

Ongoing conferences like this play a crucial role in fostering collaboration, disseminating new findings, and refining approaches to advance diagnosis, treatment, and care for individuals affected by DSAD and ADAD. They serve as essential platforms for addressing emerging challenges and opportunities in AD research and therapeutic development. With the second edition confirmed for May 2025, this conference is establishing itself as a key forum for the global scientific and medical community. The strong engagement from attendees and the meaningful contributions to scientific progress highlight the importance of maintaining this event as a recurring fixture in the research calendar. The meeting concluded with a collective commitment from participants to reconvene, building on the discussions from this year to sustain momentum in the study and treatment of DSAD and ADAD.

## CONFLICT OF INTEREST STATEMENT

Instituto de Salut Carlos III supported L.M.B. and N.F. and co‐funded by the European Union through the Río Hortega Fellowship “CM23/00291” (L.M.B.) and Juan Rodés grant JR22/00014 (N.F.). N.S.R. acknowledges support from the UK Dementia Research Institute at UCL through UK DRI Ltd, principally funded by the UK Medical Research Council, the UK NIHR UCLH Biomedical Research Centre, and the Dominantly Inherited Alzheimer Network, funded by the National Institute on Aging. L.M. is funded by grants from the “Fondation Jérôme Lejeune”, the “Agence Nationale de la Recherche (ANR)” (DYRK‐DOWN, TRANSBIOROYAL and KINHIB‐DIAB projects), France 2030 (i‐Nov vague 9, Leucettinib‐21 project), and Bpifrance (EUROSTARS, T2DiaCURE project), the European Union's Horizon 2020 research and innovation program (GO‐DS21 project) and the European Innovation Council (EIC) Accelerator Program (DOWN‐AUTONOMY project, 190138295). Alzheimer's Association ISTAART Conference Support Award. H.M.S. is a full‐time employee of the Alzheimer's Association, and their spouse is an employee at Abbott Labs in an unrelated field. L.V.A. was supported by Instituto de Salud Carlos III through the Sara Borrell Postdoctoral Fellowship “CD23/00235”. M.C.I. acknowledges support from the Alzheimer's Association and Global Brain Health Institute (GBHI_ALZ‐18‐543740), the Jérôme Lejeune Foundation (#1913 Cycle 2019B), the Societat Catalana de Neurologia (Premi Beca Fundació SCN 2020). I.R.B. was supported by Instituto de Salud Carlos III through the Río Hortega Fellowship “CM22/00052” and co‐funded by the European Union. J.E.A.I. was supported by Instituto de Salud Carlos III through the Río Hortega Fellowship “CM22/00219” and co‐funded by the European Union. J.A. was supported by Instituto de Salud Carlos III through the Río Hortega Fellowship “CM21/00243” and co‐funded by the European Union. L.M.B. was supported by Instituto de Salud Carlos III through the Río Hortega Fellowship “CM23/00291” and co‐funded by the European Union. A.B. acknowledges support from Instituto de Salud Carlos III through the Miguel Servet grant “CP20/00038” and co‐funded by the European Union, and the Alzheimer's Association “AARG‐22‐923680.” L.D.H.S. acknowledges support from Instituto de Salud Carlos III through the Miguel Servet grant CP24/00112 and co‐funded by the European Union, and the the Jérôme Lejeune Foundation (2326 ‐ GRT‐2024A). The conference was funded by grants from the Alzheimer's Association through the International Society to Advance Alzheimer's Research and Treatment (ISTAART) Grant Program for Conferences and Convenings (IGPCC) and the Pasqual Maragall Foundation, with the support of the pharmaceutical industry. J.F. reported receiving personal fees for service on the advisory boards, adjudication committees, or speaker honoraria from AC Immune, Adamed, Alzheon, Biogen, Eisai, Esteve, Fujirebio, Ionis, Laboratorios Carnot, Life Molecular Imaging, Lilly, Lundbeck, Novo Nordisk, Perha, Roche, Zambón, and outside the submitted work. D.A., A.L., and J.F. reports holding a patent for markers of synaptopathy in neurodegenerative disease (licensed to ADx, EPI8382175.0). S.G. reported receiving personal fees for service on the advisory boards, speaker honoraria, or educational activities from Esteve, Indorsia, and Biojen. MCI reported receiving personal fees for service on the advisory boards, speaker honoraria, or educational activities from Esteve, Lilly, Neuraxpharm, Adium, and Roche. Author disclosures are available in the Supporting Information.

## Supporting information



Supporting Information

## Footnotes

1 The Chairs of the conference were: Juan Fortea and Raquel Sánchez‐Valle, assisted by the members of the DSAD PIA executive committee: Beau Ances, Lisi Flores, Sigan Hartley, Florencia Iulita, Matthew Janicki, Katherine Koenig, Patrick Lao, Laurent Meijer, Michael Rafii, as well as Johannes Levin, a recognized international ADAD expert, and Heather Snyder from the Alzheimer Association.
